# Evaluation of a panel of tumor-specific differentially-methylated DNA regions in *IRF4*, *IKZF1* and *BCAT1* for blood-based detection of colorectal cancer

**DOI:** 10.1186/s13148-020-00999-y

**Published:** 2021-01-21

**Authors:** Graeme P. Young, Erin L. Symonds, Hans Jørgen Nielsen, Linnea Ferm, Ib J. Christensen, Evelien Dekker, Manon van der Vlugt, Rosalie C. Mallant-Hent, Nicky Boulter, Betty Yu, Michelle Chan, Gregor Tevz, Lawrence C. LaPointe, Susanne K. Pedersen

**Affiliations:** 1 Flinders Health and Medical Research Institute, Flinders University, Adelaide, Australia; 2 Bowel Health Service, Flinders Medical Centre, Bedford Park, South Australia Australia; 3 Department of Surgical Gastroenterology, Hvidovre Hospital, University of Copenhagen, Hvidovre, Denmark; 4 Department of Gastroenterology and Hepatology, Amsterdam University Medical Centers, Amsterdam, The Netherlands; 5 Department of Gastroenterology, Flevo Hospital, Almere, The Netherlands; 6 Clinical Genomics Pty Ltd, North Ryde, NSW Australia; 7 Clinical Genomics Technologies Ltd, Edison, USA

**Keywords:** Methylated circulating tumor DNA, *BCAT1*, *IKZF1*, *IRF4*, Colorectal cancer screening

## Abstract

**Background:**

Differentially-methylated regions (DMRs) are characteristic of colorectal cancer (CRC) and some occur more frequently than common mutations. This study aimed to evaluate the clinical utility of assaying circulating cell-free DNA for methylation in *BCAT1, IKZF1* and *IRF4* for detection of CRC.

**Methods:**

A multiplexed real-time PCR assay targeting DMRs in each of the three genes was developed. Assay accuracy was explored in plasma specimens banked from observational cross-sectional trials or from volunteers scheduled for colonoscopy or prior to CRC surgery.

**Results:**

1620 specimens were suitable for study inclusion including 184 and 616 cases with CRC and adenomas, respectively, and 820 cases without neoplasia (overall median age, 63.0 years; 56% males). Combining the PCR signals for all targeted DMRs returned the best sensitivity for CRC (136/184, 73.9%, 95% CI 67.1–79.7), advanced adenomas (53/337, 15.7%, 95% CI 12.0–20.1) and high-grade dysplastic (HGD) adenomas (9/35, 25.7%, 95% CI 14.0–42.3) with a 90.1%, specificity for neoplasia (739/820, 95% CI 87.9–92.0, *p* < 0.01). Detection of methylation in all three genes were more likely in CRC cases than those without it (OR 28.5, 95% CI 7.3–121.2, *p* < 0.0001). Of the 81 positive cases without neoplasia, 62 (76.5%) were positive by a single PCR replicate only and predominantly due to detection of methylated *BCAT1* (53.2%). Single replicate positivity was significantly higher than that in CRC (26/136, 19.1%, *p* < 0.0001), and single *BCAT1* replicate positivity was more likely in cases without neoplasia than in CRC (OR 17.7, 95% CI 6.6–43.3, *p* < 0.0001). When a positive result was limited to those with ≥ 1 PCR replicate positive for either *IKZF1* or *IRF4*, or at least two replicates positive for *BCAT1*, the multi-panel test maintained a high sensitivity for CRC (131/184, 71.2%, 95% CI 64.3–77.3) and HGD adenomas (8/35, 22.9%, 95% CI 11.8–39.3, *p* = 0.029) but improved specificity significantly (772/820, 94.1%, 95% CI 92.3–95.6, *p* < 0.0001 vs. any PCR replicate positive).

**Conclusion:**

The multi-panel methylation assay differentiates cases with CRC from those without it and does so with high specificity when criteria for *BCAT1* detection are applied. The marker panel is flexible and studies in those at average risk for CRC are now warranted to determine which panel configuration best suits screening goals.

*Trial registration*: ACTRN12611000318987. Registered 25 March 2011, https://www.anzctr.org.au/ ACTRN12611000318987.

## Introduction

Screening programs for colorectal cancer (CRC) are near universal in developed countries, but suboptimal participation rates are commonly reported, especially for stool-based screening tests [1]. A blood test might overcome the behavioral barriers observed with stool-based screening tests [2].

Circulating tumor DNA (ctDNA) is a promising biomarker of cancer, including CRC [3, 4], but reliable detection of ctDNA is subject to the frequency of the targeted tumor-specific sequence(s), whether or not the tumor DNA enters into the circulation, and to its fragmented state in circulation.

Successful implementation of a ctDNA-based test in a screening setting is technically challenging as ctDNA comprises as little as 0.01% of the total amount of circulating cell-free DNA (ccfDNA) [3]. Hence, there is a growing interest in using multiple markers for ctDNA detection to increase sensitivity for early stage neoplasia which is the ideal target for screening.

Virtually all colorectal tumors have thousands of abnormally methylated DNA regions that seem likely to be independent of various molecular sub-types of CRC [4, 5]. Many of these epigenetic changes occur more frequently and earlier in tumorigenesis than most mutations [6]. Therefore, assaying ccfDNA for CRC-specific hypermethylation in multiple regions might be a better universal identifier of CRC-derived DNA in circulation than mutations. Further, methylation-based ctDNA tests are simple in construction and not confounded by the need to cover multiple and often large regions for possible mutations.

The process of defining and validating panels of biomarkers is complex and links biomarker discovery and assay configuration with clinical validation and adaptation of a platform suitable for a clinical setting [7]. Following marker discovery and initial validation, biomarker tests will often benefit from methodological refinement to optimise assay sensitivity and specificity. We have previously reported on multiple differentially-methylated regions (DMRs) which are methylated with high frequency in CRC and the identification of DMRs residing in four genes (*BCAT1*, *IKZF1*, *IRF4* and *GRASP*) which exhibited low to no methylation in ccfDNA isolated from healthy subjects [8, 9]. An initial epigenetic ctDNA test targeting single DMRs in *BCAT1* and *IKZF1* resulted in a 62–64% sensitivity for CRC with a 92–94% specificity [10, 11]. To explore whether increasing the number of DMRs improves sensitivity for early stage CRC, we redesigned the methylation-specific ctDNA qPCR assay to include an additional DMR target in *IRF4* and changed the *IKZF1* PCR assay component to detect the targeted DMR on both strands [11, 12].

This study evaluated performance of the multi-panel assay for presence of targeted methylated regions in biobanked specimens obtained from a cohort where clinical phenotype was colonoscopy-confirmed by exploring the ways in which the targeted regions methylated with high frequency in colorectal neoplastic tissues might be utilized for blood-based detection of CRC.

## Materials and methods

### Study overview

This study was performed retrospectively using stored plasma samples that were collected from cases scheduled for colonoscopy (indicated by a wide range of clinical indications including screening and symptoms) or prior to colonic surgery. Circulating cell-free DNA (ccfDNA) was isolated from plasma, bisulphite converted and the resulting DNA was assayed in triplicate using a real-time multiplexed PCR for detection of DNA methylation in *BCAT1*, *IKZF1* and *IRF4*. The qPCR assay also targeted a region in *ACTB* as a quality control for bisulphite converted DNA. True- and false-positive results, using findings at colonoscopy as the diagnostic standard, were determined for various combinations of the three genes.

### The biobanked samples

Specimens were from plasma biobanks established from observational, predominantly prospective, cross-sectional trials undertaken at Flinders Medical Centre, Bedford Park, South Australia; Amsterdam University Medical Centers at Amsterdam Medical Center and Flevo Hospital, Almere, The Netherlands; and Hvidovre Hospital, Hvidovre, Denmark.

The Danish plasma biobank received specimens from volunteers participating in the Danish National CRC screening program. Diagnostic information was available for those undergoing colonoscopy following a fecal immunochemical test (FIT) positive result. The Dutch and Australian specimens were collected either prior to colonoscopy (for standard clinical indications including positive FIT, symptoms, surveillance due to family/personal history of neoplasia or for inflammatory bowel disease) or from cases shown at colonoscopy to have CRC and who had not yet received treatment.

An additional biobank of plasma specimens sourced through Proteogenex (CA, USA) was also included. These specimens were collected from volunteers 3–10 days after diagnostic colonoscopy (polyps were not removed) undertaken in Moscow, Russia.

The clinical trials were approved by the Institutional Review Boards of the respective sites and written informed consent was obtained from all cases for sample collection, storage and testing for research purposes. No study-wide control of colonoscopy or pathology procedures was undertaken as these specimens were collected for biomarker evaluation studies aimed to assess biomarker performance relative to outcomes determined in usual clinical practice. All venous blood was collected in either K_2_- or K_3_ EDTA tubes and processed to plasma using a 2-spin centrifugation approach (1500–3000 g for 10 min, 4–25 °C, lowest deceleration setting).

### Clinical classification and specimen selection

Clinical phenotype was determined using clinicopathological findings by experts at each site, with main outcomes categorized as CRC, adenoma or no neoplasia. CRC was further subcategorized into stages according to the AJCC 7th Edition [13]. Advanced adenoma was defined as adenoma with any of the following characteristics: (a) ≥ 10 mm in size, (b) villous histology (> 20% villous component), (c) high-grade dysplasia (HGD) and/or (d) the presence of ≥ 3 tubular adenomas (< 10 mm and with low-grade dysplasia (LGD). The presence of multiple adenomas was included with the advanced adenoma classification as previous studies have shown that these lesions are associated with an increased risk for future advanced neoplasia [14, 15]. Non-advanced adenoma refers to those not meeting the characteristics of an advanced adenoma. Adenomas were also classified separately into dysplasia status. Stage 0 CRC, where there was severe cellular atypia or marked architectural distortion but no evidence of invasion, was included in advanced adenoma (as HGD). The most advanced neoplasm was used as the principal diagnosis when multiple colorectal pathologies were present.

Specimens were selected for inclusion in the analysis on the basis of sufficient plasma available for testing (3.9–4.5 mL) provided that complete clinical and demographic data were available. Cases excluded were those with known or suspected cancer of another organ at the time of collection, familial adenomatous polyposis or hereditary non-polyposis CRC syndrome (Lynch syndrome) or incomplete diagnostic information.

### Detection of methylated DNA in plasma

All frozen plasma samples were couriered to Clinical Genomics Technologies for storage and subsequent testing (Sydney, NSW, Australia). ccfDNA was isolated from plasma using the QS DSP Circulating DNA Kit (Qiagen) on a QIASymphony SP instrument as per manufacturer’s instruction (Qiagen) and bisulphite converted using the EpiTect Fast 96 DNA Bisulfite Conversion kit (Qiagen) on a QIACube HT instrument as previously described [11]. The resulting purified bisulphite-converted DNA (~ 45μL) was assayed as triplicates of 12μL in a total PCR volume of 30μL on a Light Cycler 480 II (Roche Diagnostics, IN, USA) as previously described [11] (see Additional file 1: Fig. S1 for further details). Cycle threshold (Ct) values were calculated using the second derivative maximum algorithm provided with the LC480 software. The *ACTB* assay component was used for estimation of ccfDNA yield as well as a quality control parameter. Samples with mean *ACTB* Ct values ≥ 36.6 were not accepted for analysis unless positive for one or more of the methylation targets. Plasma specimens were processing by staff who were blinded to the associated clinical and demographic data.

### Statistical methods

Detection rates for each DMR were determined for each clinical phenotype and assay positivity was based on using various combinations of the DMRs. Sensitivity for a colorectal neoplastic condition was estimated from the positivity rate in the presence of that clinical phenotype. Specificity for neoplasia was estimated from the positivity rate in the absence of neoplasia. Medians and interquartile ranges (IQR) were determined where appropriate. Clinical sub-populations were compared using two correlated proportion methodologies for discrete (*Z* score two-population proportion test and Chi Square test, or Fisher’s exact test when sample size was small) and continuous data (Wilcoxon rank-sum test). One-way ANOVA was used to compare three or more independent populations. McNemar’s test was used for concordance analyses. Multivariable logistic regression was used to explore the impact of gender, age, other comorbidities and yield on assay result. All statistical tests were two-sided and a *p* value of < 0.05 determined statistical significance. All analyses were performed using GraphPad Prism version 8.2.0 as well as the online tool (http://graphpad.com/scientific-software).

## Results

### Study population

In total, 1620 specimens were suitable for assay and obtained from patients that met the inclusion criteria. Patient characteristics for each of the four collection sites are shown in Table 1.

**Table 1:** Demographic characteristics and clinical findings by collection site

	*N* (%)	AUS	DEN	NLD	RUS
		*n* (%)			
Cases	1620 (100)	643 (39.7)	774 (47.8)	101 (6.2)	102 (6.3)
Males	902 (55.7)	367 (57.1)	435 (56.2)	59 (58.4)	41 (40.2)^a^
Median age (min–max)	63.0 years (18.1–88.0)	62.6 years (18.1–85.4)	64.3 years (50.0–75.8)	62.0 years (37.0–88.0)	56.5 years^a^ (34.0–86.0)
Cancer	184 (11.4)	27 (14.7)	91 (49.5)	17 (9.2)	49 (26.6)
Males	97 (52.7)	14 (51.9)	52 (57.1)	10 (58.8)	21 (42.9)
Median age (min–max)	67.2 years (35.0–88.0)	66.2 years (45.7–81.1)	68.7 years (50.1–75.2)	70.0 years (37.0–88.0)	62.0 years^a^ (35.0–86.0)
Stage I	41 (22)	7 (17.1)	20 (48.8)	3 (7.3)	11 (26.8)
Stage II	57 (31)	9 (15.8)	20 (35.1)	7 (12.3)	21 (36.8)
Stage III	51 (28)	5 (9.8)	35 (68.6)	4 (7.8)	7 (13.7)
Stage IV	33 (18)	6 (18.2)	16 (48.5)	3 (9.1)	8 (24.2)
Unstaged	2 (1%)	0 (–)	0 (–)	0 (–)	2 (100)
Adenoma	616 (38.0)	332 (53.9)	197 (32.0)	52 (8.4)	35 (5.7)
Males	387 (62.8)	207 (62.3)	131 (66.5)	33 (63.5)	16 (45.7)^a^
Median age (min–max)	63.5 years (33.7–85.4)	64.1 years (33.7–85.4)	64.1 years (50.0–75.8)	62.4 years (51.0–75.0)	56.1 years^a^ (34.0–82.0)
Advanced	337 (54.7)	147 (44.3)	149 (75.6)	38 (73.1)	3 (8.6)
Non-advanced	279 (45.3)	185 (55.7)	48 (24.4)	14 (26.9)	32 (91.4)
HGD^b^	35 (5.7)	15 (4.5)	18 (9.1)	2 (3.8)	0 (0)
LGD^b^	549 (89.1)	317 (95.5)	179 (90.9)	18 (34.6)	35 (100)
No neoplasia	820 (50.6%)	284 (34.6)	486 (59.3)	32 (3.9)	18 (2.2)
Males	418 (51.0)	146 (51.4)	252 (51.9)	16 (50.0)	4 (22.2)^a^
Median age (min–max)	60.0 years (18.0–85.0)	56.7 years (18.0–85.0)	62.2 years (50.0–75.0)	61.6 years (52.0–75.0)	47.5 years^a^ (36.0–59.0)

AUS, Flinders Medical Centre, Australia; DEN, Hvidovre Hospital, Denmark; NLD, Amsterdam University Medical Centers or Flevo Hospital, The Netherlands; RUS, Proteogenex

^a^Chi square, collection site significantly different from the other sites

^b^The severity of dysplasia was available for 584 of 616 adenomas. *HGD * high grade dysplasia, *LGD* low grade dysplasia

The specimens included 184 cases with CRC [median 67.2 years (35–88), 52.7% males], 616 with adenomas [median 63.5 years (33.7–85.4), 62.8% males] and 820 cases without neoplasia [median 60.0 years (18.0–85.0), 51.0% males]. Compared to the other three collection sites, the specimens from Proteogenex had a lower content of males and were obtained from patients 5–6 years younger.

### Detection of methylated DNA by clinical phenotype

**Fig. 1:**
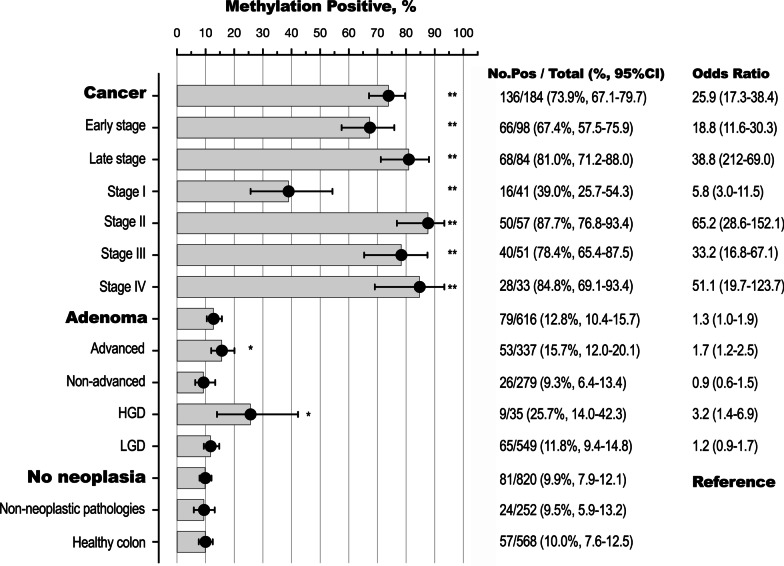
Detection of methylated DNA by clinical phenotype. A specimen was deemed positive (Pos) if at least one PCR replicate was positive for DNA methylation in at least one of the 3 genes. Black closed circles, calculated mean detection rates (%); horizontal bars, 95% CI; severity of dysplasia was available for 584 of 616 adenomas, *HGD *high grade dysplasia, *LGD* low grade dysplasia; non-neoplastic pathologies included benign polyps (hyperplastic, unspecified, inflammatory, other polyps), inflammatory bowel disease, diverticular disease, angiodysplasia, hemorrhoids; Odds ratios (95% CI) are relative to cases without neoplasia, **p < 0.0001; *p < 0.05. Two of the 184 CRC cases were unstaged [2/2, 100% (15.8–100)] and were omitted from the figure

Of the 1620 plasma specimens, 296 had at least one PCR replicate (18.3%, 95% CI 16.4–20.2) positive for methylation in any one of the three genes. The detection rate was highest in those with CRC (136/184, 73.9% sensitivity), followed by a 15.7% detection rate for advanced adenomas (53/337), which was also significantly higher than that of non-advanced adenomas, 9.3% (26/279, *Z *score *t *test *p* = 0.018) and those without neoplasia [81/820, 9.9% (specificity 90.1%), *p* = 0.005], Fig. 1.

When the multi-target assay was positive for methylation in any one of the targeted genes, the odds ratio for presence of CRC was high compared to cases without neoplasia [odds ratio (OR) for CRC 25.9, 95% confidence interval (95% CI): 17.3–38.4, *p* < 0.0001], Fig. 1. The odds of advanced adenoma was also higher (OR 1.7, 95% CI 1.2–2.5, *p* = 0.006), whereas the odds ratio for non-advanced adenoma was not different from those without neoplasia (OR 0.9, 95% CI 0.6–1.5, *p* = 0.907).

The individual markers of methylation in *BCAT1*, *IKZF1* and *IRF4* also discriminated well between those with cancer or advanced adenomas, and those with no neoplasia, Table 2.

**Table 2:** Positivity rate for methylated *BCAT1, IKZF1* and *IRF4* in each clinical phenotype

	*N*	*BCAT1*		*IKZF1*		*IRF4*	
		*n*, Pos^a^ (%, 95% CI)	OR (95% CI)^b^	*n*, Pos. (%, 95% CI)	OR (95% CI)	*n*, Pos. (%, 95% CI)	OR (95% CI)
All cases	1620	173 (10.7, 9–12)	–	185 (11.4, 10–13)	–	138 (8.5, 7–9)	–
Cancer	184	87 (47.3, 40–55)	15.8 (10–24)*	109 (59.2, 52–66)	32.6 (21–51)*	92 (50.0, 43–57)	44.6 (26–77)*
Early stage	98	38 (38.8, 29–49)	11.2 (7–19)*	51 (52.0, 42–62)	24.3 (15–41)*	40 (40.8, 31–51)	30.7 (17–56)*
Late stage	84	48 (57.1, 46–68)	23.5 (14–40)*	56 (66.7, 56–77)	44.9 (26–80)*	50 (59.5, 48–70)	65.5 (35–124)*
Stage I	41	8 (19.5, 10–34)	4.3 (2–10)*	11 (26.8, 13–41)	8.2 (4–18)*	7 (17.1, 5–29)	9.2 (4–23)*
Stage II	57	30 (52.6, 40–65)	19.6 (11–35)*	40 (70.2, 58–82)	52.8 (27–99)*	33 (57.9, 45–71)	61.3 (30–122)*
Stage III	51	23 (45.1, 33–59)	14.5 (8–27)*	33 (64.7, 51–78)	41.1 (21–81)*	28 (54.9, 41–69)	54.2 (27–112)*
Stage IV	33	25 (75.8, 59–87)	55.1 (24–121)*	23 (69.7, 53–86)	51.6 (23–114)*	22 (66.7, 50–84)	89.1 (36–209)*
Unstaged	2	1 (50.0, 3.–97)	17.6 (91–335)		–*	2 (100, 16–100)	–*
Adenoma	616	42 (6.8, 5–9)	1.3 (1–2)	41 (6.7, 5–9)	1.6 (1–3)	28 (4.5, 3–6)	2.1 (1–4)*
Advanced	337	29 (8.6, 6–12)	1.7 (1–3)*	29 (8.6, 6–12)	1.7 (1–3)*	20 (5.9, 3–9)	2.8 (2–5)*
Non-advanced	279	13 (4.7, 3–8)	0.7 (0.5–2)	12 (4.3, 2–7)	1.0 (0.5–2)	8 (2.9, 1–5)	1.3 (0.6–3)
HGD^c^	35	5 (14.3, 6–30)	2.9 (1–8)*	6 (17.1, 8–33)	4.6 (2–12)*	7 (20.0, 10–36)	11.1 (4–29)*
LGD^c^	549	35 (6.4, 5–9)	1.2 (0.7–1.9)	32 (5.8, 4–8)	1.4 (1–2)	18 (3.3, 2–5)	1.5 (0.8–3)
No Neoplasia	820	44 (5.4, 4–7)	Ref	35 (4.3, 3–6)	Ref	18 (2.2, 1–3)	Ref
Non-neoplastic pathologies^d^	252	14 (5.6, 3–8)	–	9 (3.6, 1–6)	–	10 (4.0, 2–6)	–
Healthy colon	568	30 (5.3, 3–7)	–	26 (4.6, 3–6)	–	8 (1.4, 0.4–2)	–

^a^At least one PCR replicate positive for DNA methylation

^b^Odds ratio (95% CI) compared to cases without neoplasia (Ref), *p values < 0.05

^c^The severity of dysplasia was available for 584 of 616 adenomas; HGD, high grade dysplasia; LGD, low grade dysplasia

^d^Benign polyps (hyperplastic, unspecified, inflammatory, other polyps), inflammatory bowel disease, diverticular disease, angiodysplasia, hemorrhoids

### Positivity rate by stage of neoplasia

**Fig. 2:**
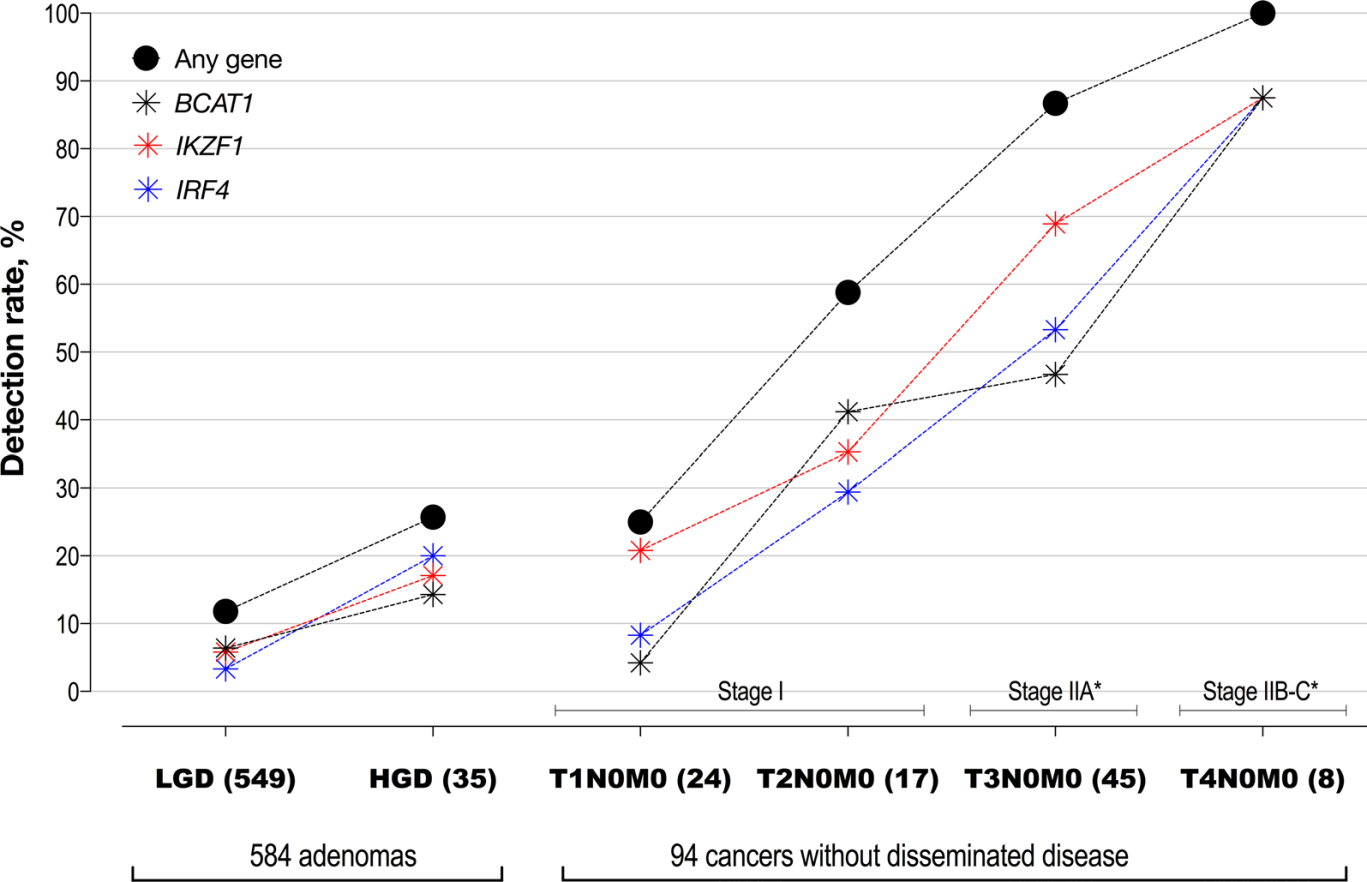
Positivity by progression of cellular atypia (dysplasia in adenomas) and degree of invasion (T stage in cancer), in cases without disseminated disease. *LGD *low grade dysplasia, *HGD *high grade dysplasia. Y axis: average detection rates, % (sensitivity). Closed black circles—at least one PCR replicate positive for methylation in any of the three genes; blue, red and black symbols—detection rates of the individual methylation markers, *IRF4*, *IKZF1* and *BCAT1*, respectively. Additional file 1: Table S1 provides details for count of positives, % detected and 95% CI. *Only 53 of the 57 Stage II cases had full TNM information

The detection rate of methylated DNA increased significantly from non-advanced adenoma to stage IV CRC (Fig. 1; one-way ANOVA, post-test for trend, *p* < 0.0001). The increase by stage was also observed for each of the targeted genes, Table 2. The detection rate (sensitivity) for CRC increased significantly with increasing depth of invasion as assessed by T-stage (Fig. 2, one-way ANOVA *p* < 0.0001). The detection rate in Stage I T2N0M0 cancers was more than two fold higher than that for Stage I T1N0M0 (*p* = 0.029).

When classifying adenomas by grade of dysplasia, the detection rate was significantly higher for those with HGD (9/35, 25.7%, 95% CI 14.0–42.3) than for those with LGD (65/549, 11.8% (9.4–14.8), *p* = 0.017), Fig. 2 and Additional file 1: Table S1. Adenomas with HGD and T1N0M0 cancers returned similar detection rates, 25.7% and 25.0%, respectively. The odds of adenoma with HGD being present given a positive multi-target assay was high compared to no neoplasia (OR 3.2, 95% CI 1.4–6.9, *p* = 0.007). The odds of an adenoma with LGD was not different from those with no neoplasia (OR 1.2, 95% CI 0.9–1.7, *p* = 0.284). These positivity patterns were mirrored in each of the targeted three genes, Fig. 2 and Additional file 1: Table S1.

### Concordance between methylated genes

**Fig. 3:**
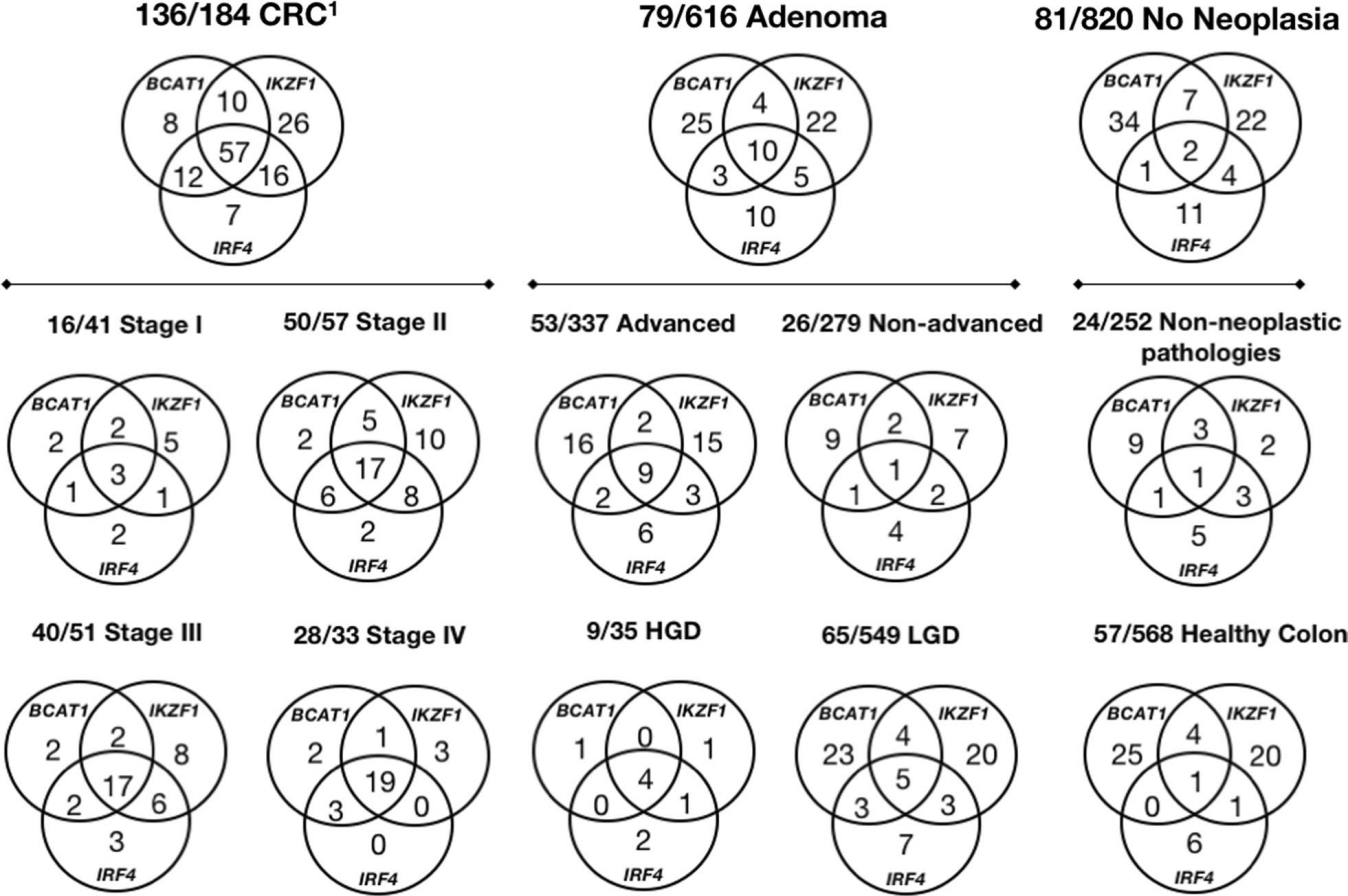
Concordance between positive methylated *BCAT1*, *IKZF1* and *IRF4* results in plasma. The number of positive specimens is shown for each phenotype, together with the numbers returning a positive for each *BCAT1*, *IKZF1* and *IRF4* combination.^1^There were two unstaged cancer cases, both positive for methylation, which are not included in the CRC Stage Venn diagrams. Severity of dysplasia was available for 584 of 616 adenomas, *HGD *high grade dysplasia, *LGD* low grade dysplasia; non-neoplastic pathologies included benign polyps (hyperplastic, unspecified, inflammatory, other polyps), inflammatory bowel disease, diverticular disease, angiodysplasia, hemorrhoids

Of the 296 specimens positive for at least one of the biomarkers, 69 (23.3%) were positive for methylation in all three targeted genes (i.e. concordance was positive), Fig. 3. The proportion of cases positive for methylation in all three genes was highest for CRC (57/136 positives, 41.9%) and lowest for cases without neoplasia (2/81 positives, 2.5%). When the multi-target assay was positive for DNA methylation in all 3 genes, the odds ratio for presence of cancer compared to those with no neoplasia was 28.5 (95% CI 7.3–121.2, *p* < 0.0001).

The proportion of CRC cases positive for methylation in any of the targeted genes rose with increasing stage: Stage I—3/16 (18.8%), Stage II—17/50 (34.0%), Stage III—17/40 (42.5%), Stage IV—19/28 (67.9%), ANOVA post trend test, *p* = 0.005. Similarly, the three genes were positive in 4/9 (44.4%) adenomas with HGD compared to 5/65 (7.7%; *p* = 0.01, Fisher’s exact test) with LGD.

### Test performance refinement based on different methylation target combinations

Each of the targeted methylated regions contributed to the detection of cancer (true-positives), and different combinations of these targets are shown in Table 3. The best specificity, 97.8%, was achieved using *IRF4* as the sole marker of methylated DNA in circulation with a 50% sensitivity. The best sensitivity (73.9%) was achieved using all three genes with a specificity of 90.1%. A similar trend was observed in advanced adenomas and adenomas with HGD, with the best sensitivity for these being achieved using all three genes, Additional file 1: Table S2.

**Table 3:** Test accuracy for detection of colorectal cancer based on all possible gene combinations

Gene combination	Counts		Sensitivity % (95% CI)	Specificity % (95% CI)	AUC (95% CI)	LRP (95% CI)
	TP	TN				
*BCAT1* only	87	776	47.3 (40.2–54.5)	94.6 (92.9–96.0)	0.710 (0.662–0.757)	8.8 (6.4–12.2)
*IRF4* only	92	802	50.0 (42.8–57.2)	97.8 (96.6–98.6)	0.739 (0.691–0.787)	22.8 (14.1–36.8)
*IKZF1* only	109	785	59.2 (52.0–66.1)	95.7 (94.1–96.9)	0.775 (0.730–0.820)	13.9 (9.8–19.6)
*BCAT1* and-or *IRF4*	110	761	59.8 (52.6–66.6)	92.8 (90.8–94.4)	0.763 (0.718–0.808)	8.31 (6.3–10.9)
*IKZF1* and-or *IRF4*	128	773	69.6 (62.6–75.8)	94.3 (92.5–95.7)	0.819 (0.778–0.860)	12.1 (9.1–16.3)
*BCAT1* and-or *IKZF1*	129	750	70.1 (63.1–76.3)	91.5 (89.4–93.2)	0.808 (0.767–0.849)	8.21 (6.4–10.5)
Any of the 3 genes	136	739	73.9 (67.1–79.7)	90.1 (87.9–92.0)	0.820 (0.781–0.859)	7.48 (6.0–9.4)

The true- and false-positive rates in cases with cancer (*n* = 184) and cases without neoplasia (*n* = 820) were used for sensitivity (for cancer) and specificity (for neoplasia) estimates

*TP* Counts of true positives, *TN* Counts of true negatives, *AUC* area under the curve of ROC plots shown in Fig. 5, *LRP* positive likelihood ratio

Of the 81 false-positive cases without neoplasia, 67 (82.7%) were positive for methylation in a single gene only, and methylation in *BCAT1* was the most frequent cause of the three genes (34/67, 50.7%).

### PCR replicate positivity by clinical status

**Fig. 4:**
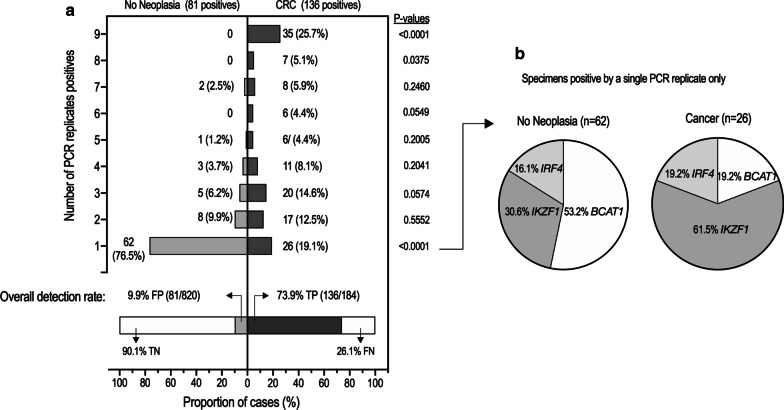
PCR replicate positivity rates in positive specimens. **a** Frequency distribution of the number of PCR replicates positive for methylation in patients without neoplasia (*n* = 81) and with cancer (*n* = 136). Each assayed specimen generates a total of 9 PCR replicates, 3 for each gene. **b** In cases (cancer or no neoplasia) where only one replicate was positive, the proportion for each gene responsible for the positive result. *FP* false positive, *TP* true positive, *TN* true negative, *FN* false negative

In view of the effect of *BCAT1* detection on specificity and as a total of nine PCR replicate results were generated (three for each gene target) per assayed specimen, the relationship between PCR replicate count and clinical status was examined. A comparison of PCR replicate positivity between cases with CRC and those without neoplasia showed that the number of positive PCR replicates was much higher in cases with CRC, Fig. 4. Of the 136 CRC cases with a DNA methylation signal (i.e. at least 1 PCR replicate positive for methylation in either *BCAT1*, *IKZF1* and/or *IRF4*), 35 (25.7%) cases were methylation positive in all 9 PCR replicates and 26 (19.1%) were methylation positive by just a single PCR replicate. In contrast, none of the 81 positive cases without neoplasia returned 9 positive PCR replicates. Of the 67 cases without neoplasia that were positive for methylation in a single gene only, 62 (92.5%) were positive by just a single PCR replicate. Single PCR replicate positivity was significantly higher than that in CRC (62/81, 76.5% vs. 26/136, 19.1%, *Z *score *p* < 0.0001).

### Evaluation of a replicate rule for panel positivity

**Fig. 5:**
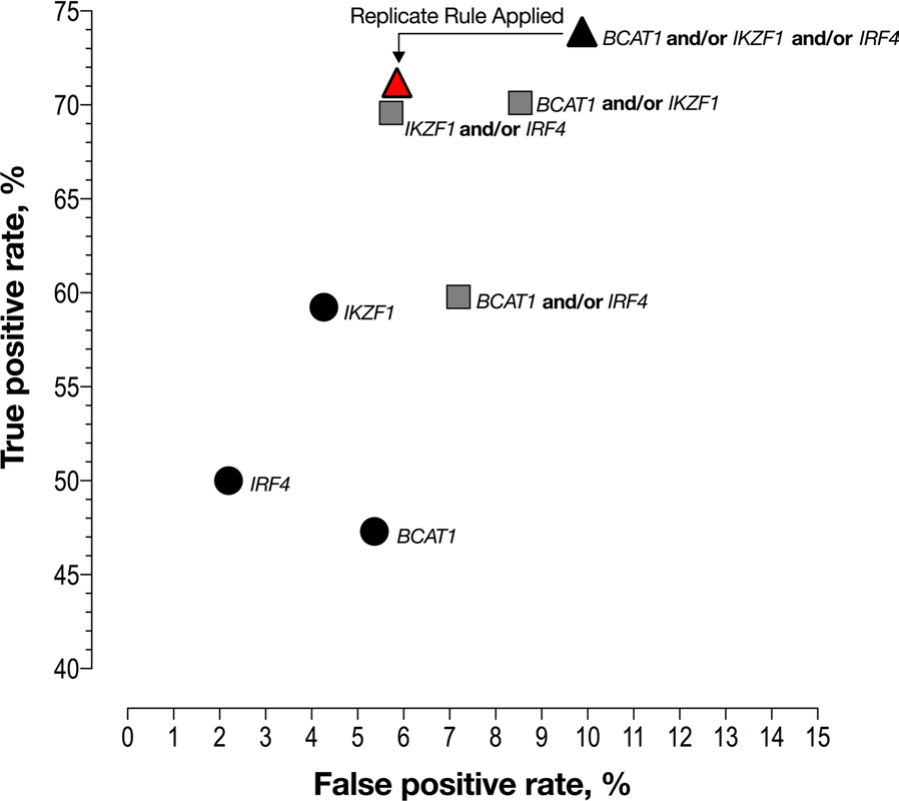
ROC analysis for potential combinations of different genes for cancer detection. Black dots—one methylation target; grey squares—two methylation targets; black triangle—all three methylation targets. Red triangle: resulting performance specification when applying the ‘*BCAT1* replicate rule’ to the 3-gene combination (see text for details)

More than half of the single PCR replicate positive cases without neoplasia were due to detection of methylation in *BCAT1* (33/62, 53.2%, Fig. 4b). As the odds ratio for a single *BCAT1* positive PCR replicate representing a false-positive (i.e. a case without neoplasia) compared to a true-positive (i.e. a case with CRC) was 17.7 (95% CI6.6–43.3), *p* < 0.0001), a ‘*BCAT1* replicate rule’ for assay positivity requiring at least two positive *BCAT1* replicates was evaluated. For the ‘*BCAT1* replicate rule’, a specimen was deemed positive when at least one PCR replicate was positive for methylation in either *IKZF1* or *IRF4*, or at least two PCR replicates were positive for methylation in *BCAT1*. With this rule, the false-positive rate for cancer was significantly reduced from 9.9% (81/820) to 5.9% (48/820, 95% CI4.4–7.8, *p* < 0.0001), while the sensitivity was not significantly affected; 73.9% (136/184, 95% CI 67.1–79.7) versus 71.2% (131/184, 95% CI 64.1–77.6; *p* = 0.562). To explore the dynamic nature of assay response to number of replicates, a ROC curve was created that yielded an area under the curve (AUC) of 0.827 (0.786–0.867) and the LRP was 12.2 (9.1–16.3). The odds ratio for presence of CRC compared to those without neoplasia was 25.9 (95% CI 17.3–38.4) for the multi-target assay (any gene positive) compared to 39.8 (95% CI 25.7–61.5) if using the ‘*BCAT1* replicate rule’. Figure 5 compares ROC analysis of possible combinations of genes and the effect of applying the ‘*BCAT1* replicate’ rule for detection of CRC.

When applying the replicate rule, the true-positive rates (sensitivity) for detection of advanced adenomas compared to a three gene panel without the rule was significantly lower (53/337, 15.7%, 95% CI 12.2–20.0 vs. 37/337, 11.0%, 95% CI 8.1–14.8, *p* = 0.048); as was the case for adenomas with HGD (9/35, 25.7%, 95% CI 14.0–42.3 vs. 8/35, 22.9%, 95% CI 11.8–39.3, *p* = 0.029). Additional file 1: Table S2.

### Confounding variables associated with assay positivity in cases without neoplasia

A variety of non-malignant pathologies, comorbidities and demographic factors, such as age and gender, are known to be associated the detection of methylated DNA in blood [16–18]. Logistic regression was used to explore the impact of clinical variables on assay positivity in the subgroup without neoplasia, Table 4. Yield of ccfDNA was a significant independent predictor of multi-target assay positivity in cases without neoplasia (OR 1.89, 95% CI 1.30–2.75, *p* = 0.0008). Male gender and aging showed a positive association with assay positivity but failed to reach significance (*p* = 0.07–0.10).

**Table 4:** Predictors of test positivity (any marker detected) in cases without neoplasia

Predictor	Odds ratio^a^	95% CI	*p* value
Gender (male)	1.57	0.97–2.59	0.072
Log(ccfDNA Yield)	1.89	1.30–2.75	0.0008
50–59 years of age	1.00	0.35–3.29	0.993
60–69 years of age	1.97	0.74–6.25	0.206
70–79 years of age	2.30	0.84–7.47	0.128
Over 80 years of age	3.82	0.69–18.40	0.098
Colorectal non-neoplastic pathologies^b^	1.26	0.65–2.45	0.492
Other comorbidities^c^	1.50	0.84–2.72	0.170

^a^Odds ratios are estimated for each covariate independently. 95% confidence intervals are shown for odds ratio

^b^Benign polyps (hyperplastic, unspecified, inflammatory, other polyps), inflammatory bowel disease, diverticular disease, angiodysplasia, hemorrhoids

^c^Hypertension, diabetes, asthma, chronic ischemia, angina pectoris, atherosclerosis, cerebral infarction, myocardial infarction, rheumatoid arthritis, chronic obstructive pulmonary disease

As the yield of ccfDNA was the only independent predictor of assay positivity in cases without neoplasia, we looked at known sources of ccfDNA variance [19, 20]. There was a significant relationship between yield of ccfDNA and increasing age (*p* = 0.0001), inflammatory disorders (*p* = 0.008) and male gender (*p* = 0.015).

Including all 1620 specimens, ccfDNA levels varied greatly (median 3.8 ng/mL, IQR 2.5–5.5) but were only significantly elevated in cases with stage III (4.7 ng/mL, IQR 2.6–9.1) or IV (5.6 ng/mL, IQR 3.8–9.9) cancer compared to cases with no evidence of disease (3.6 ng/mL, IQR 2.4–5.2, *p* < 0.0001).

## Discussion

Following initial discovery of potential biomarkers for the detection of CRC, the next step is to determine the efficient and effective biomarker combinations that are best able to discriminate between those with and without cancer. This evaluation is an important step before moving to translational research in a screening context [21]. We have previously reported on genes, including *BCAT1* and *IKZF1*, that contain regions methylated with high frequency in neoplastic colonic lesions and detection of such DMRs in DNA in circulation for indication of CRC [10, 11]. In an endeavor to achieve better sensitivity, we developed a multi-target qPCR assay detecting an additional DMR in *IRF4* as well as detecting an additional DMR in *IKZF1*. The resulting multi-target qPCR assay was evaluated in a population comprising the spectrum of pathologies typically encountered when screening for CRC and which was more diverse than the cohort used in our initial discovery [10].

We found that each of the targeted DMRs residing in *BCAT1*, *IKZF1* and *IRF4* contributed to detection of CRC and advanced adenomas (especially those with HGD) with a significantly higher odds ratio for either disease state being present. The multi-target qPCR assay discriminated between CRC or advanced adenomas compared to those without neoplasia and was more sensitive than previously observed when just 2 DMRs were included.

Positive concordance between the three genes was highest in those with cancer and also higher for those with HGD adenomas compared to cases without neoplasia. Overall, this concordance reflects an increasing rate of aberrant methylation as neoplasia progresses.

As expected, increasing the number of targets resulted in a lower specificity than previously observed [10, 11]. Detection of circulating DNA methylated in only *BCAT1* was the most prevalent event leading to a false-positive assay result.

The assay positivity rate increased as one progressed from adenoma through to late stage cancer indicating that presence of hypermethylated tumor DNA in plasma was dependent on stage of cancer, especially T stage, but also on degree of dysplasia (the initial morphological change characterizing neoplasia) in adenomas. Adenomas with HGD were detected at a similar rate to T1N0M0 CRC, which was a significantly higher rate than those with non-advanced adenomas or adenomas with LGD. The detection rate of advanced adenomas (defined by commonly accepted clinical criteria) was significantly higher than the detection rate of those with non-advanced adenomas but significantly lower those with HGD. This positivity pattern is not unexpected since the clinical definition for advanced adenoma include states that relate to risk of developing metachronous neoplasia at a later stage and are not restricted to morphological features characterising progressive cellular atypia at the time of testing for the methylation biomarkers. These findings for HGD adenomas are novel and point to potential for using this class of ctDNA biomarkers to target clinically relevant adenomas as well as cancer.

The relationship between marker detection and cancer stage agrees with previous observations, which indicates that as neoplastic lesions progress and especially as they invade, the number of ctDNA molecules increases due to egress into the circulation [22, 23]. Each of the targeted ctDNA methylation markers had a 39–52% sensitivity for early-stage CRC (Table 2) which aligns with performance reported for other single-target somatic- or epigenetic-based ctDNA tests [3, 24]. Enhancing the analytical sensitivity of the assay by targeting just 44 methylated CpG sites in 4 DMRs residing in 3 genes (Figure S1) resulted in a 67.4% sensitivity for early stage CRC which is comparable to the 60.4% reported sensitivity for early stage CRC using a ctDNA blood test targeting more than 28,000 DMRs [25].

This study demonstrates that, with the right biomarkers, it is possible to design a relatively simple ctDNA test that can detect CRC with good sensitivity without the need for large dimensional data sets such as those involving next generation sequencing. Importantly, our strategy avoids the inflexibility of complex marker algorithms and shows that by using different marker combinations, an end-user has flexibility to adjust the assay output to suit the clinical goals (especially important in screening), such as maximising detection (sensitivity) while controlling cost-effectiveness (specificity) or feasibility. For instance, if a high specificity is required but a 50% sensitivity for CRC is acceptable, as is already the case in some screening programs around the world that use a conservative fecal haemoglobin concentration cut-off for FIT [26], this is achievable by detection of just methylated *IRF4* DNA in blood alone, although this approach would require further validation in a true screening context. But other jurisdictions consider a higher sensitivity for CRC, as observed with the multitarget assay here, as being desirable. The risk of adding biomarkers is that specificity falls as one strives for higher sensitivity. By considering the specificity of each individual biomarker, however, we have been able to improve specificity of the panel from 90.1% to 94.1% with little compromise in sensitivity for CRC (73.9% vs. 71.2%). This improvement was achieved by defining a specimen positive for methylation if there was at least one PCR replicate positive for methylation in *IKZF1* or *IRF4* (irrespective of *BCAT1* positivity), or at least two PCR replicates positive for methylation in *BCAT1* (the “replicate rule”). Using this rule, the odds ratio for presence of CRC was 39.8 when positive for the rule compared to an OR of 25.9 for the full biomarker panel when not applying the replicate rule. This improvement was because *BCAT1* was the major contributor to deterioration in specificity. A high replicate count was characteristic of patients with CRC (a quarter of cancer cases were positive in all nine replicates) while in the majority of cases without neoplasia, only one replicate was positive, usually *BCAT1*. Based on the cohort tested herein, the odds ratio of a single *BCAT1* positive PCR replicate being a false-positive compared to a true positive was 17.7.

The three targeted genes examined in this study may play a role in the tumorigenesis of CRC. Differential methylation in the *BCAT1* promoters alters the ratio of generated BCAT1 protein isoforms [27], and aberrant expression of BCAT1 has been associated with CRC [28]. The promoters of *IKZF1* and *IRF4* are silenced when hypermethylated [29, 30]. The transcriptional factors, IKZF1 and IRF4 are important transcriptional regulators of *notch* and *c-myc* [29, 31–33], and c-MYC have been linked to BCAT1 expression [34]. As well-described for c-MYC, both IKZF1 and IRF4 are involved in the development of cancer, including CRC [29, 35, 36].

This study has strengths and limitations. As the estimates of test accuracy are derived from biobanked specimens from four sites where patients were undergoing diagnostic assessment by colonoscopy for a wide range of clinical applications, the actual accuracy estimates might not be the same in an unbiased typical screening population. Thus, it will be important to proceed with application of the panel described here in a prospective population screening study, ideally compared with another proven screening test such as the fecal immunochemical test. The diverse nature of the population, however, has advantages given the broad spread of colorectal pathologies. We were able to comprehensively assess the impact of non-neoplastic conditions and other possible confounding variables associated with detection of these biomarkers that might affect specificity in a large number of subjects. The concentration of ccfDNA was the only significant factor affecting the multi-target assay response in the subgroup of cases without neoplasia. Male gender and those aged over 80 years showed a weak positive association with assay result but failed to reach significance.

As observed by others, the levels of ccfDNA varied greatly in cases without neoplasia [18, 37–39], and there was a significant relationship between higher ccfDNA concentrations and increasing age.

When screening for CRC, it is ideal to use a test capable of detecting advanced adenomas given that their removal reduces incidence of CRC. Thus, endoscopic screening for CRC, or use of high-sensitivity FIT (with sensitivity for advanced adenomas in the order of 40%), is adopted in some jurisdictions. By targeting multiple regions methylated with high frequency and at the earliest onset of colorectal neoplasia, we have achieved a sensitivity of 25% for adenomas exhibiting HGD which raises the prospect of further success with future modifications of the panel.

Similar observations of increased adenoma detection have been observed for the well-studied methylation biomarker, *SEPT9* when studied in a small cohort of 76 cases in conjunction with another methylation marker, *ALX4*. The additional of *ALX4* increased sensitivity for advanced precancerous colorectal lesions from 12 to 45% (6/49 vs. 22/49) [24, 40] compared to *SEPT9* alone. Nevertheless, the specificity in that study also fell significantly from 95.5% (1/22) to 82% (4/22) with the additional biomarker demonstrating that the higher positivity was not specific for neoplasia. The present study did not suffer such a large increase in false positives.

Our findings show that detection of methylated *BCAT1*, *IKZF1* and *IRF4* in circulating ccfDNA differentiates cases with CRC from those without neoplasia, and that the specificity of the multi-target assay can be substantially improved with no significant effect on sensitivity by applying a PCR replicate rule to *BCAT1*. This panel of markers should now be prospectively evaluated in a typical screening population to clarify accuracy of different marker configurations against that of FIT. As there is considerable global variation in what constitutes acceptable sensitivity and/or specificity [1], the flexibility which is provided by using different configurations will allow health care provides to choose a performance that suits the goals of specific screening programs.

## Supplementary Information

**Additional file 1: Figure S1**. The multi-panel real-time PCR asssay. **Table S1**. Positivity by progression of cellular atypia (dysplasia in adenomas) and degree of invasion (T stage in cancer) in cases without disseminated disease. **Table S2**. Test accuracy for detection of advanced adenomas and adenomas with high grade dysplasia based on all possible combinations of genes.

## Data Availability

The dataset analyzed during this study are available from the corresponding author on reasonable requests.
