# Supplementary material for: Evaluation of a panel of tumor-specific differentially-methylated DNA regions in IRF4, IKZF1 and BCAT1 for blood-based detection of colorectal cancer
Source: Clin Epigenetics. 2021 Jan 21;13:14. doi: 10.1186/s13148-020-00999-y (PMC7818774; doi:10.1186/s13148-020-00999-y)
Supplement: Supplementary file 1 — Additional file 1: Figure S1. The multi-panel real-time PCR asssay. Table S1. Positivity by progression of cellular atypia (dysplasia in adenomas) and degree of invasion (T stage in cancer) in cases without disseminated disease. Table S2. Test accuracy for detection of advanced adenomas and adenomas with high grade dysplasia based on all possible combinations of genes. [file 13148_2020_999_MOESM1_ESM.pdf]

Additional File

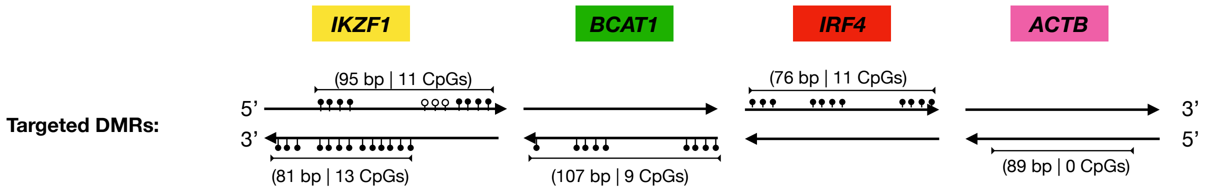

Figure S1. The multi-panel real-time PCR assay. (A) Schematic representation of the investigated multi-panel qPCR assay, which targets differentially-methylated regions (DMRs) in *BCAT1* ((Branched Chain Amino Acid Transaminase 1), *IKZF1* (IKAROS Family Zinc Finger 1) and *IRF4* (Interferon Regulatory Factor 4) and a control region in *ACTB* (beta-actin). The *IKZF1* PCR assay component detects the targeted DMR on both strands (non-complementary subsequent to bisulphite conversion). Partially methylation in the *IKZF1* DMR residing on the sense strand was detectable by utilization of degenerate probes. The *IKZF1* probes were all labelled with the same fluorophore, FAM. Detection of the other amplicon targets were reported using probes labelled with HEX (*BCAT1*), Texas Red (*IRF4*) and Cy5 (*ACTB*).

Table S1. Positivity[1] by progression of cellular atypia (dysplasia in adenomas) and degree of invasion (T stage in cancer) in cases without disseminated disease.

	N	Any gene	BCAT1	IKZF1	IRF4
			Count of Positives, n (%, 95%CI)		
**584 adenomas[2]**					
HGD	35	9 (25.7, 14-42)	5 (14.3, 6-29)	6 (17.1, 4-30)	7 (20.0, 6-34)
LGD	549	65 (11.8, 9-15)	35 (6.4, 5-9)	32 (5.8, 4-8)	18 (3.3, 2-5)
P-value[3]		0.017	0.072	0.009	<0.0001
**94 cancers without disseminated disease[4]**					
T1N0M0	24	6 (25.0, 6-44)	1 (4.2, -5-13)	5 (20.8, 55-83)	2 (8.3, -4-20)
T2N0M0	17	10 (58.8, 33-85)	7 (41.2, 15-67)	6 (35.3, 10-61)	5 (29.4, 5-54)
T3N0M0[5]	45	39 (86.7, 76-97)	21 (46.7, 32-62)	31 (68.9, 55-83)	24 (53.3, 38-69)
T4N0M0	8	8 (100, 63-100)	7 (87.5, 58-117)	7 (87.5, 58-117)	7 (87.5, 6-117)
P-value[6]		<0.0001	<0.0001	<0.0001	<0.0001

[1] At least one PCR replicate positive for DNA methylation in any gene.

[2] The severity of dysplasia was available for 584 of 616 adenomas.

[3] Z-score population t-test.[4] T stage in cancer cases without disseminated disease.

[5] 57 Stage II (T3N0M0 and T4N0M0) in study cohort, but only 53 had full TNM information.

[6] Ordinary one-way ANOVA against T1N0M0.

Table S2. Test accuracy[1] for detection of advanced adenomas and adenomas with high grade dysplasia based on all possible combinations of genes.

Gene combination	TP	Sensitivity % (95%CI)	AUC (95%CI)	TN	Specificity % (95%CI)
**Advanced adenoma (N = 337):**					
BCAT1 only	29	8.6 (6.0-12.1)	0.516 (0.479-0.553)	776	94.6 (92.9-96.0)
IRF4 only	20	5.9 (3.8-9.0)	0.503 (0.467-0.540)	802	97.8 (96.6-98.6)
IKZF1 only	29	8.6 (6.0-12.1)	0.516 (0.479-0.553)	785	95.7 (94.1-96.9)
BCAT1 and-or *IRF4*	38	11.3 (8.3-15.1)	0.530 (0.492-0.567)	761	92.8 (90.8-94.4)
IKZF1 and-or *IRF4*	37	11.0 (8.0-14.8)	0.528 (0.491-0.565)	773	94.3 (92.5-95.7)
BCAT1 and-or *IKZF1*	47	14.0 (10.6-18.1)	0.543 (0.505-0.580)	750	91.5 (89.4-93.2)
Any of the 3 genes	53	15.7 (12.2-20.0)	0.552 (0.514-0.589)	739	90.1 (87.9-92.0)
Application of BCAT1 Replicate Rule:					
Any of the 3 genes	37	11.0 (8.1-14.8)	0.526 (0.488-0.563)	772	94.1 (92.3-95.6)
**Adenoma with high-grade dysplasia (N = 35):**					
BCAT1 only	5	14.3 (5.8-29.9)	0.545 (0.441-0.648)	776	94.6 (92.9-96.0)
IRF4 only	7	20.0 (9.7-36.2)	0.573 (0.467-0.679)	802	97.8 (96.6-98.6)
IKZF1 only	6	17.1 (7.7-33.1)	0.559 (0.454-0.664)	785	95.7 (94.1-96.9)
BCAT1 and-or *IRF4*	8	22.9 (11.8-39.3)	0.587 (0.480-0.694)	761	92.8 (90.8-94.4)
IKZF1 and-or *IRF4*	8	22.9 (11.8-39.3)	0.587 (0.480-0.694)	773	94.3 (92.5-95.7)
BCAT1 and-or *IKZF1*	7	20.0 (9.7-36.2)	0.573 (0.467-0.679)	750	91.5 (89.4-93.2)
Any of the 3 genes	9	25.7 (14.0-42.3)	0.602 (0.494-0.709)	739	90.1 (87.9-92.0)
Application of BCAT1 Replicate Rule:					
Any of the 3 genes	8	22.9 (11.8-39.3)	0.587 (0.480-0.694)	772	94.1 (92.3-95.6)

[1] The true- and false-positive rates in cases with advanced adenomas (n=337) or adenomas with high grade dysplasia (n=35) and cases without neoplasia (n = 820) were used for sensitivity (for indicated adenomas) and specificity (for neoplasia) estimates. TP: Counts of true positives. TN: Counts of true negatives. AUC, Area Under Curve.
